# Interplay between base excision repair protein XRCC1 and ALDH2 predicts overall survival in lung and liver cancer patients

**DOI:** 10.1007/s13402-018-0390-8

**Published:** 2018-08-07

**Authors:** Xin Chen, Arnaud J. Legrand, Siobhan Cunniffe, Samuel Hume, Mattia Poletto, Bruno Vaz, Kristijan Ramadan, Dengfu Yao, Grigory L. Dianov

**Affiliations:** 1 CRUK & MRC Oxford Institute for Radiation Oncology, Department of Oncology, University of Oxford, Old Road Campus Research Building, Oxford, OX3 7DQ UK; 2 Research Centre of Clinical Medicine, Affiliated Hospital of Nantong University, Jiangsu, China; 3 School of Life Science, Nantong University, Nantong, China; 4 Institute of Cytology and Genetics, Russian Academy of Sciences, Lavrentyeva 10, Novosibirsk, Russian Federation 630090; 5 Novosibirsk State University, Novosibirsk, Russian Federation 63000

**Keywords:** ALDH2, Aldehydes, DNA damage, XRCC1, Base excision repair, Genetic instability, Liver and lung carcinomas, Mithramycin a

## Abstract

**Background:**

To deliver efficacious personalised cancer treatment, it is essential to characterise the cellular metabolism as well as the genetic stability of individual tumours. In this study, we describe a new axis between DNA repair and detoxification of aldehyde derivatives with important implications for patient prognosis and treatment.

**Methods:**

Western blot and qPCR analyses were performed in relevant non-transformed and cancer cell lines from lung and liver tissue origin in combination with bioinformatics data mining of The Cancer Genome Atlas database from lung and hepatocellular cancer patients.

**Results:**

Using both biochemical and bioinformatics approaches, we revealed an association between the levels of expression of the aldehyde detoxifying enzyme aldehyde dehydrogenase 2 (ALDH2) and the key DNA base excision repair protein XRCC1. Across cancer types, we found that if one of the corresponding genes exhibits a low expression level, the level of the other gene is increased. Surprisingly, we found that low ALDH2 expression levels associated with high XRCC1 expression levels are indicative for a poor overall survival, particularly in lung and liver cancer patients. In addition, we found that Mithramycin A, a XRCC1 expression inhibitor, efficiently kills cancer cells expressing low levels of ALDH2.

**Conclusions:**

Our data suggest that lung and liver cancers require efficient single-strand break repair for their growth in order to benefit from a low aldehyde detoxification metabolism. We also propose that the ratio of XRCC1 and ALDH2 levels may serve as a useful prognostic tool in these cancer types.

**Electronic supplementary material:**

The online version of this article (10.1007/s13402-018-0390-8) contains supplementary material, which is available to authorized users.

## Introduction

Aldehydes are abundant organic molecules that can be assimilated from food or produced within cells as by-products of cellular metabolism. Among many cellular aldehydes, acetaldehyde (AcAl) is the most studied, particularly for its importance in cancer metabolism [1–3]. Endogenous AcAl is mainly produced by the cellular processing of alcohol and also as an intermediate in sugar metabolism [4]. Aldehydes are highly reactive molecules, generating a range of DNA modification products including DNA strand crosslinks and DNA-protein crosslinks [4]. Due to their toxicity, AcAls are processed in human cells by detoxification enzymes such as aldehyde dehydrogenase 2 (ALDH2), which oxidises AcAl to acetate [5]. Until recently, very little was known about how cells respond to DNA damage induced by AcAls. Recent studies suggest that AcAl-induced DNA damage is likely to be processed by multiple DNA repair pathways due to its complexity [6–9]. Two DNA repair pathways involved in repair of DNA crosslinks and DNA double strand breaks have been implicated in the repair of AcAl-induced DNA damage thus far [6]. However, in addition to DNA strand crosslinks and DNA-protein crosslinks, AcAls also generate multiple DNA base modifications and neither of these pathways can deal with base modifications. N2-ethylidenedeoxyguanosine is the main adduct, and its reduced form N2-ethyldeoxyguanosine [10] has been directly linked to ALDH2, since its levels increase in ALDH2 knockout mice treated with ethanol [11].

Base excision repair (BER) is the primary mechanism responsible for repairing most endogenous DNA lesions, including numerous types of damages to nitrogenous bases that can be induced by AcAls [12]. BER is a well understood DNA repair pathway [13, 14]. It is initiated by a DNA glycosylase that recognizes the damaged DNA base and cleaves the N-glycosylic bond that binds the DNA base to the sugar-phosphate backbone. A new site without a base residue (also called an abasic site, an AP site or an apurinic/apyrimidinic site) is further processed by an AP endonuclease (APE1 in human cells) that cleaves the phosphodiester bond next to the AP site, thereby generating a DNA strand break containing a hydroxyl residue at the 3′-end and deoxyribose phosphate at the 5′-end. Further, DNA polymerase β, using its AP-lyase activity, removes deoxyribose phosphate from the 5′-end and simultaneously adds one nucleotide to the 3′-end of the nick. DNA repair is completed by sealing the ends by a complex of two proteins, XRCC1 and DNA ligase IIIα [12]. XRCC1 is a scaffold protein that is essential for the formation and stabilization of the ternary DNA polymerase β-XRCC1-DNA Ligase IIIα complex that is necessary to complete BER [15]. Hence, cells deficient in XRCC1 are characterized by reduced DNA repair and increased genomic instability (reviewed in [16]).

We previously showed that loss of BER triggers metabolic changes, notably by increasing the activity of the one-carbon cycle [17], which endogenously produces and metabolises formaldehyde, an even more reactive molecule than AcAl [2]. If BER-deficient cells increase the activity of this cycle, it is reasonable to suggest that this would, in turn, increase the concentration of formaldehyde in cells as both a waste product and a reaction intermediate. We thus hypothesised that BER-deficient cells may exhibit increased levels of aldehydes. As a result, these cells would be in need of increasing an aldehyde detoxification system, such as ALDH2, to counteract AcAl and formaldehyde toxicity. Here, we tested this hypothesis along with its potential consequences for cancer aggressiveness and prognosis.

## Materials and methods

### Cell cultures and drug

The non-transformed bronchial epithelium cell line LIMM-NBE1 has previously been described in [18]. The normal human fibroblast cell lines TIG-1 and WI38 were obtained from the Coriell Institute Cell Repository. The non-small cell lung carcinoma-derived cell line H1299 was obtained from the American Type Culture Collection (ATCC), while the liver carcinoma-derived cell line JHH4 was kindly provided by Prof Ricky Sharma (University College London). All cell lines were cultured in DMEM (Life Technologies) supplemented with either 15% FBS (for the LIMM-NBE1 cell line) or 10% FBS (for the cancer-derived cell lines) at 37 °C in a humidified atmosphere with 5% CO_2_. Cells were routinely checked for mycoplasma. Mithramycin A was purchased from Enzo Life Sciences.

### siRNA transfection

siRNA transfections were carried out using the Lipofectamine RNAiMAX reagent (Life Technologies) according to the manufacturer’s protocol. Unless otherwise indicated, cells were transfected with 30 nM siRNA and analysed 72 h after transfection. siRNA oligonucleotides were obtained from Eurogentec. Sequences were as follows:

- XRCC1 5’-AGGGAAGAGGAAGUUGGAU-3′, TDP1 5’-GACCAUAUCUAGUAGUGAU-3′ and ALDH2 5’-CCUCAAAUGUCUCCGGUAU-3′. Control transfections were carried out using a non-targeting siRNA (Eurogentec, SR-CL000–005), referred to as NC.

### Western blotting

Whole cell extracts for Western blotting were prepared as described previously [19]. The antibodies used were directed against XRCC1 (MS-1393-P0, Neomarkers), ALDH2 (102–10,056, Cambridge Biosciences), β-actin (ab6276, Abcam) and α-Tubulin (T6199, Sigma Aldricht). Detection and quantification was carried out using an Odyssey image analysis system (Li-Cor Biosciences).

### DNA-protein crosslink isolation and quantification

LIMM-NBE1 cells were grown to 100% confluency in 10-cm dishes in order to synchronise them in the G1 phase, as XRCC1 siRNA stops the cell cycle in this phase. DNA-protein crosslinks (DPCs) were detected using a modified rapid approach to the DNA adduct recovery assay [20]. In brief, 1.5 to 2 × 10^6^ cells were lysed in 1 ml M buffer (MB) containing 6 M Guanidine Thiocyanate, 10 mM Tris–HCl (pH 6.8), 20 mM EDTA, 4% Triton X100, 1% Sarkosyl and 1% Dithiothreitol. DNA was precipitated by adding 1 ml 100% ethanol and washed three times in wash buffer (20 mM Tris–HCl pH 6.8, 150 mM NaCl and 50% ethanol) after which DNA was solubilized in 1 ml 8 mM NaOH. A small aliquot of the recovered DNA was digested with 50 μg/ml proteinase K (Invitrogen) for 3 h at 50 °C and quantified using PicoGreen dye (Invitrogen) according to the manufacturer’s instructions. The DNA concentration was further confirmed by slot-blot analysis followed by immunodetection with an antibody directed against dsDNA. To quantify DPCs, DNA was digested with benzonase (Invitrogen) for 30 min at 37 °C, after which proteins were precipitated by the standard Trichloroacetic Acid protocol [21], dissolved in Laemmli buffer, resolved by SDS-PAGE and visualized by silver staining.

### Immunofluorescence assay

Immunofluorescence was carried out following standard procedures. Briefly, cells were fixed with paraformaldehyde (4% in PBS for 15 min). Permeabilisation was carried out using Triton X-100 (0.2% in PBS for 10 min at 4 °C) after which cells were saturated with 5% bovine serum albumin (BSA) in PBS for 1 h. Subsequent incubation with an anti-ALDH2 antibody (102–10,056, Cambridge Biosciences) was carried out in 5% BSA-PBS supplemented with 0.01% Tween 20. Alexa Fluor 594-conjugated secondary antibodies (Life Technologies) were used for indirect detection of the antigens. Hoechst 33,342 (Life Technologies) was used to visualise nuclei. Images were taken by confocal microscopy using a Zeiss LSM 710 microscope.

### Quantitative real-time PCR (qRT-PCR)

Total RNA was extracted using a RNeasy kit (Qiagen) and cDNA was prepared using a SuperScript RT-PCR system (Life Technologies) as per manufacturer’s instructions. qRT-PCR was performed using a Fast SYBR® Green Master Mix (Applied Biosystems) according to the manufacturer’s protocol. Reactions were carried out using a 7500 Fast Real-Time PCR System (Applied Biosystems). The comparative CT method was applied for quantification of gene expression. GAPDH and B2M were used as endogenous controls, unless otherwise stated. The following primers were used:

- ALDH2 (For: CCTCTCCAGTGGACGGATT; Rev.: CGAGGTCTTCTGCAACCAG)
- GAPDH (For: AGCCACATCGCTCAGACAC; Rev.: GCCCAATACGACCAAATCC)
- B2M (For: ATGTCTCGCTCCGTGGCCTTA; Rev.: ATCTTGGGCTGTGACAAAGTC)
- TBP (For: CGGTTTGCTGCGGTAATCAT; Rev.: TTTCTTGCTGCCAGTCTGGAC)
- XRCC1 (For: CTGGGACCGGGTCAAAAT; Rev.: CAAGCCAAAGGGGGAGTC)
- APE1 (For: CGGACAAGGAAGGGTACAGT; Rev.: CAAATTCAGCCACAATCACC)
- TDP1 (For: CGCTTGTTTCTTCAGCTCAG; Rev.: ACAAGCAGGATTGGCTTCTT)

### Clonogenic assay

500 cells were seeded per well in 6-well plates, with three technical replicates of each variable. In Figs. 8D and E, siRNA transfections were carried out 24 h before seeding. In Fig. 9A, cells were allowed to attach for at least 18 h before Sp1 inhibitor addition, at the indicated concentrations. Sp1 inhibitor was added for in total 48 hours, replenishing the inhibitor at 24 h. Surviving cells were allowed to proliferate for 10–14 days. Cells were fixed for 30 min in 0.4% methylene blue dye in methanol. Only colonies of more than 50 cells were counted using the automated system GelCount™ by Oxford Optronix. Each graph represents the mean of at least 3 independent experiments.

### Bioinformatics analyses

Bioinformatics analyses were performed using publicly available data from the Oncomine® (www.oncomine.org), cBioPortal (http://www.cbioportal.org) [22, 23] and TCGA (RNAseq data) (cancergenome.nih.gov) databases. Studies using the Oncomine® database can be found in Table S1. All data extracted from cBioPortal (originally from TCGA) can be found in Table S2 (Lung cancer [NSCLC]), Table S3 (Liver cancer) and Table S4 (Esophageal cancer).

### Statistical analyses

Statistical analyses were performed by the two-tailed Student’s t-test using either Microsoft Excel or SPSS (IBM).

## Results

### ALDH2 expression is increased following BER deficiency

**Fig. 1:**
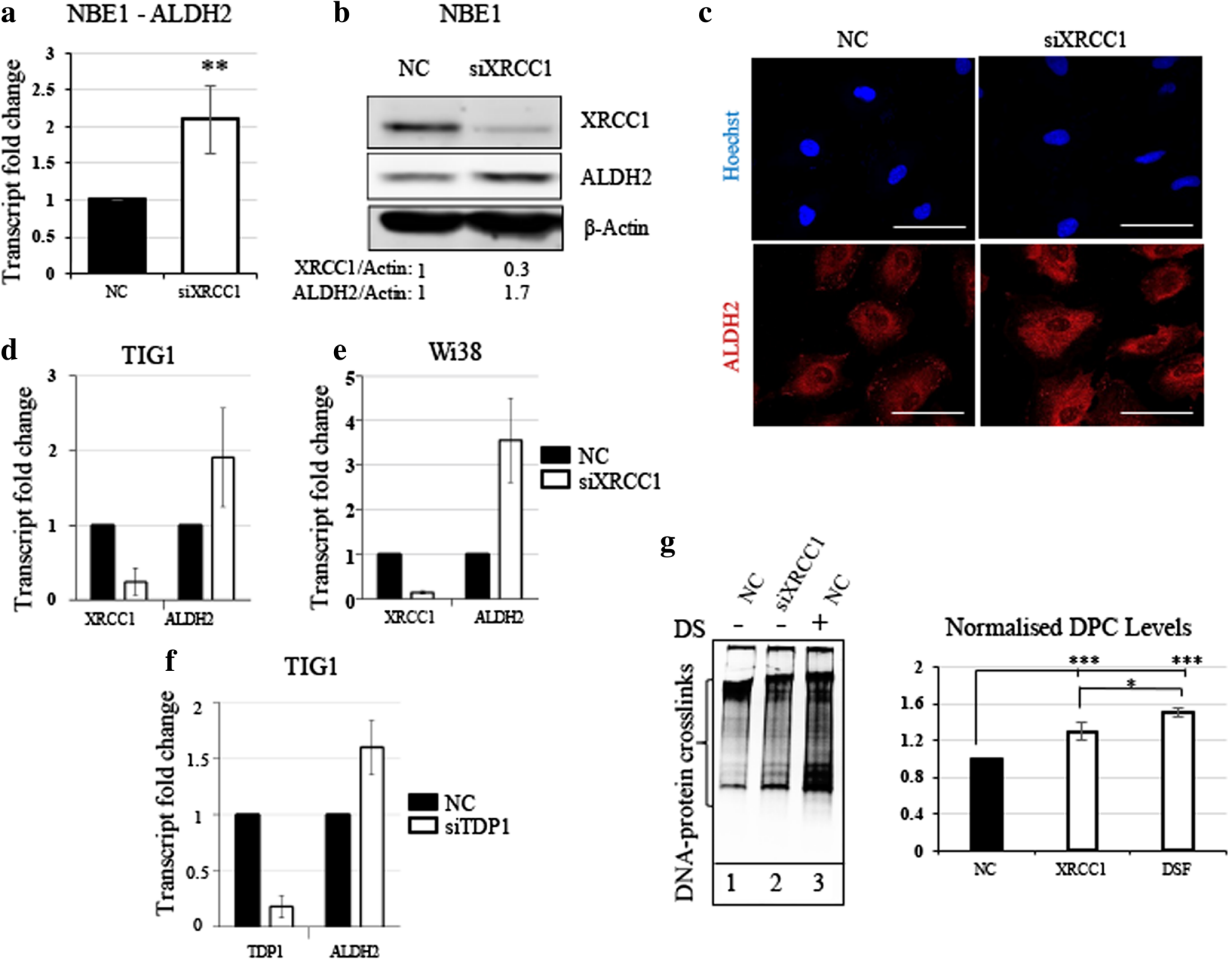
**XRCC1 depletion causes an increased expression of ALDH2 and increased DNA-protein crosslinks. a** qRT-PCR analysis of ALDH2 transcript levels in NBE1 cells after 72 h of XRCC1 knock down (KD). **b** Representative Western blot analysis of ALDH2 and XRCC1 levels in NBE1 cells after 72 h of XRCC1 KD. **c** Representative immunofluorescence analysis of ALDH2 in NBE1 cells after 72 h of XRCC1 KD. **d** qRT-PCR analysis of ALDH2 and XRCC1 transcript levels in TIG1 cells after 72 h of XRCC1 KD. **e** qRT-PCR analysis of ALDH2 and XRCC1 transcript levels in WI38 cells after 72 h of XRCC1 KD. **f** qRT-PCR analysis of ALDH2 and TDP1 transcript levels in TIG1 cells after 72 h of TDP1 KD. qRT-PCR reference genes are B2M and GAPDH for a, d and e, and TBP and GAPDH for f. **g** Left panel, representative silver staining analysis of protein crosslinked onto DNA in NBE1 cell extracts after 72 h of XRCC1 KD or 24 h of disulfiram treatment (DS, 10 μM). Right panel, densitometric quantification of the data. Data are expressed as mean ± SD from at least three independent experiments * *p* < 0.05; ** *p* < 0.01; *** *p* < 0.001

We previously showed that BER-deficient cells adjust their metabolism to prevent oxidative stress by producing anti-oxidant molecules such as GSH [17]. We hypothesised that BER-deficient cells operate in a similar way to prevent aldehyde-induced DNA damage by enhancing detoxification systems. To test this, we downregulated BER in the non-transformed cell line LIMM-NBE1 (hereafter NBE1) by reducing the expression of the key BER protein XRCC1, using siRNA. We observed a significant increase in ALDH2 expression both at the mRNA (Fig. 1A) and protein (Fig. 1B) level 72 h after siRNA treatment. The localisation of ALDH2, as shown by immunofluorescence, was unchanged and the increase corresponds to a cytoplasmic localisation (Fig. 1C). The increase in ALDH2 expression following XRCC1 depletion was also observed in two additional normal human fibroblast cell lines TIG1 (Fig. 1D) and WI38 (Fig. 1E). APE1, POLβ and XRCC1 are essential components of BER. However, APE1 has diverse cellular functions and its depletion is highly toxic, creating many types of lesions, ranging from apurinic/apyrimidinic AP-sites to double-strand breaks [24, 25]. POLβ knockout cells are BER deficient, but to a lesser extent than XRCC1 knockout cells [26]. We therefore considered XRCC1 knockdown as an optimal model of BER deficiency [17]. Nevertheless, it is also possible to simulate BER deficiency by creating unresolved single-strand breaks (SSBs) through the depletion of BER end processor protein tyrosyl-DNA phosphodiesterase 1 (TDP1), which catalyses the removal of Type I topoisomerase blocked at the 3-prime phosphate end of the DNA single strand break [17]. Doing so, we again observed an increase in ALDH2 expression (Fig. 1F). This would suggest that it is the accumulation of SSBs that triggers ALDH2 up-regulation. Next, we investigated whether this balancing mechanism was linked to the detoxification of aldehydes and their products, DNA-protein crosslinks (DPCs [27]). Despite an increased amount of ALDH2, we observed a slight but significant increase in DPCs in XRCC1-deficient cells compared to control cells treated with unspecific siRNA (Fig. 1G, compare lanes 1 and 2). This might suggest that even with more ALDH2, DPCs are still occurring more frequently in BER-deficient cells. In positive control experiments, inhibition of aldehyde detoxification by the pan-ALDH inhibitor Disulfiram led to substantial DPC accumulation in NBE1 cells (Fig. 1G, lane 3), confirming that ALDH2 detoxification activity can prevent DPC formation. From these results, we conclude that BER-deficient cells accumulate SSBs that might directly or indirectly, by increasing for example the amount of reactive aldehydes [26], provoke an increase in DPCs. In response, cells may increase ALDH2 expression, potentially in order to prevent further accumulation of DPCs.

### ALDH2 expression is low in most cancers, whereas XRCC1 expression is high

**Fig. 2:**
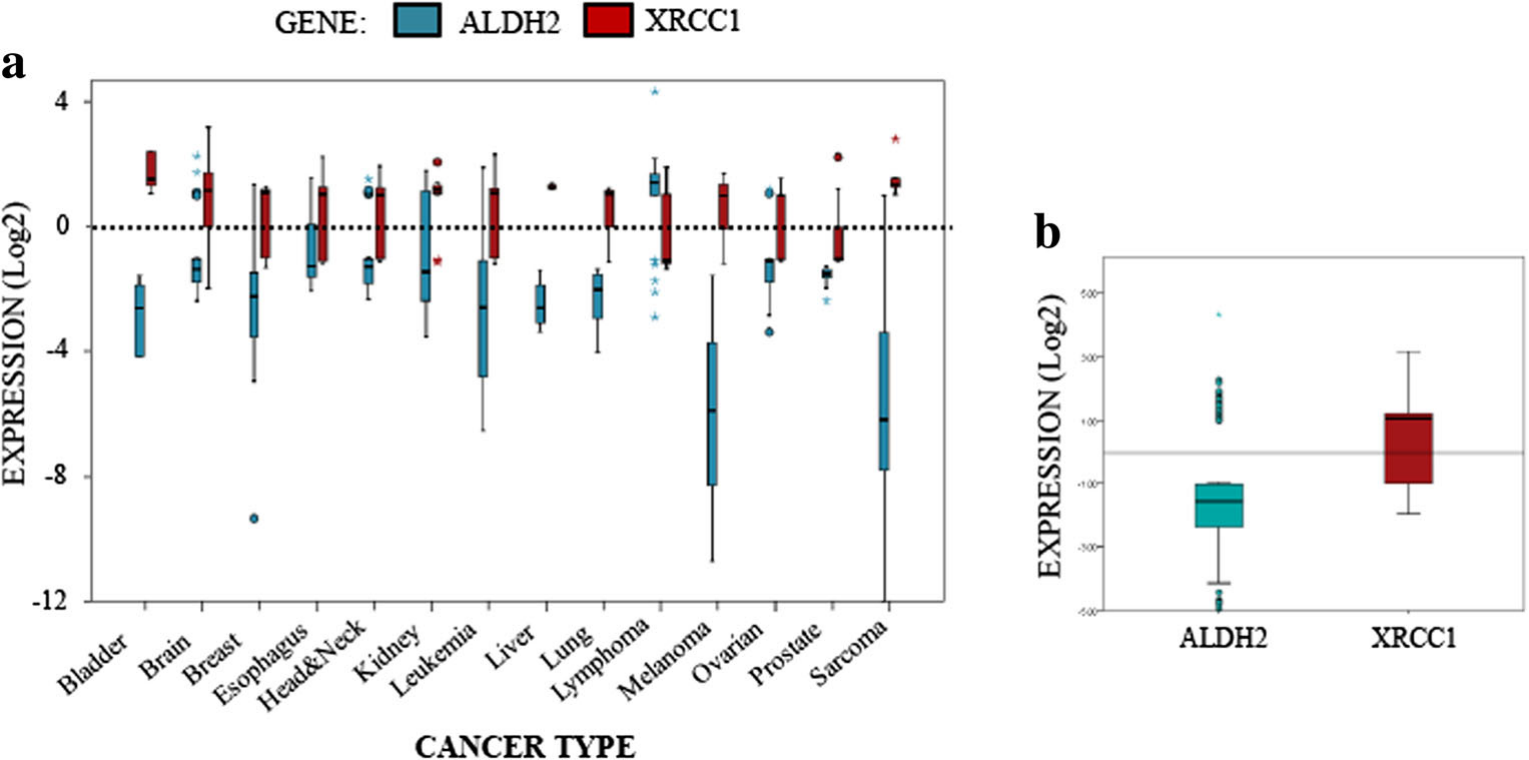
**XRCC1 and ALDH2 mRNA levels mirror each other in most cancer types. a** Boxplot summarising the fold changes in mRNA levels for ALDH2 (blue) and XRCC1 (red) depending on the cancer type. **b** Boxplot representing the overall variation in XRCC1 and ALDH2 mRNA levels in all cancer types. A list of the studies used and the associated mRNA fold changes can be found in Table S1

DPCs are a major threat to DNA replication and genomic stability. Accumulation of DPCs has been observed during aging, neurodegeneration and cancer development [28]. In order to understand why cancer cells are unable to prevent the induction of DPCs and other DNA damage, caused by the accumulation of reactive aldehydes, we carried out bioinformatics analyses of ALDH2 mRNA levels in different cancer types using the Oncomine® database (Fig. 2). Strikingly, we noticed that, when ALDH2 mRNA expression is significantly deregulated, it is for a major part by downregulation (Fig. 2A and B). We found that the levels of ALDH2 mRNA are particularly low in melanoma, sarcoma, leukaemia and bladder cancer (Fig. 2A). It is also important to emphasize that ALDH2 mRNA is expressed at its highest levels in tissues such as liver, brain and lungs [29]. All of these tissues, when cancerous, showed lower ALDH2 mRNA levels (Fig. 2A). Interestingly, we found that the XRCC1 mRNA expression levels seem to generally reverse ALDH2 mRNA expression levels by being higher in most cancerous tissues (Fig. 2A and B). This is more obvious in sarcoma and bladder cancer, but even more interestingly in liver, brain and lung cancers (Fig. 2A). Globally at the median value, we found that ALDH2 is downregulated by more than two-fold and XRCC1 is upregulated by more than two-fold across all cancer types (Fig. 2B). Altogether, these analyses suggest that the increase in DPCs in cancer cells might be caused by a lack of ALDH2 expression. This deficiency, however, coincides with a higher expression of XRCC1, which is required for the BER pathway.

### Low ALDH2 levels predict a poor 5-year overall survival rate for lung and liver cancers, but not for oesophagus cancer

**Fig. 3:**
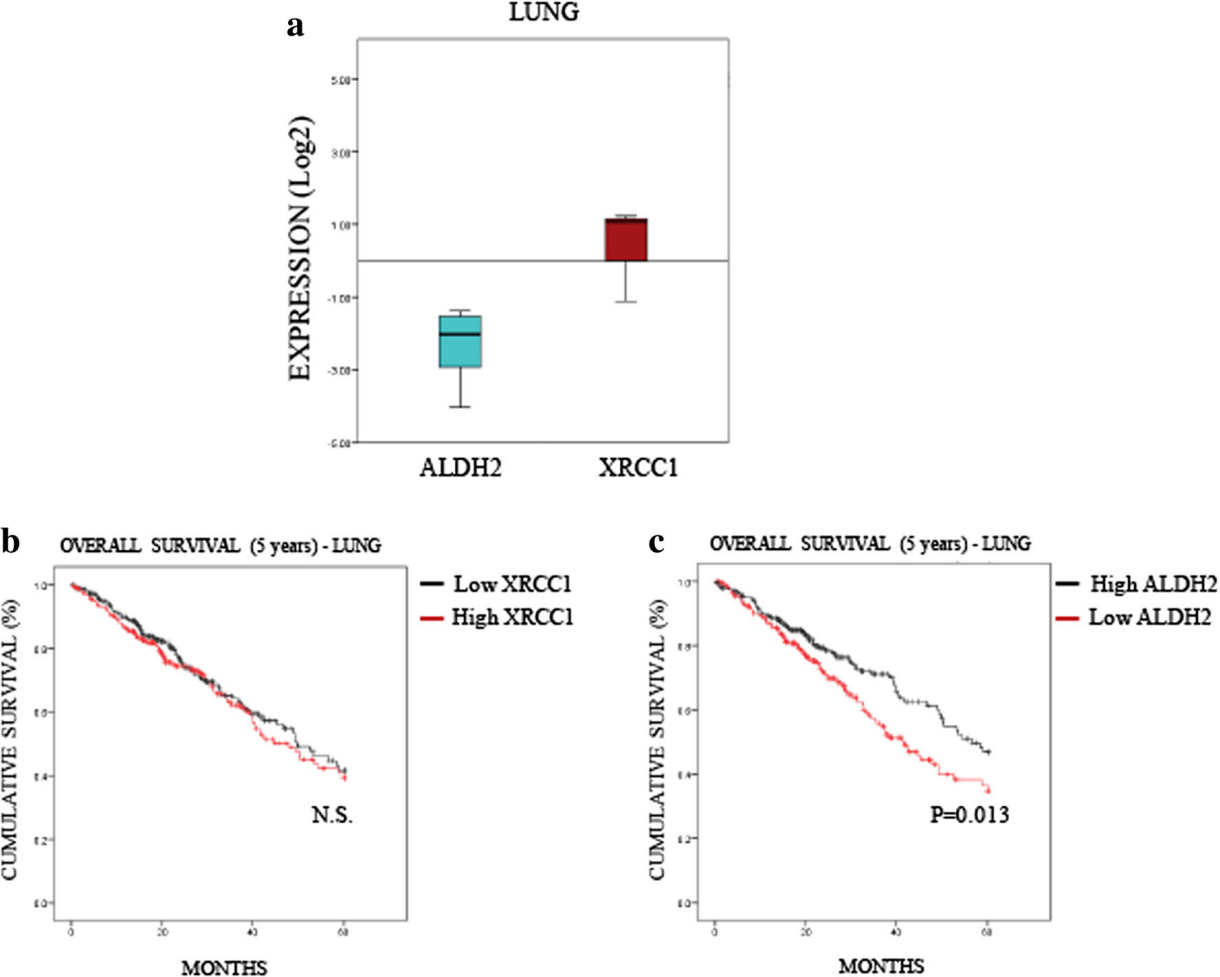
**Low ALDH2 expression predicts a poor prognosis in lung cancer. a** Boxplot representing the overall variation in XRCC1 and ALDH2 mRNA levels in lung cancers (Oncomine). **b** Kaplan-Meier analysis of the 5-year overall survival of lung cancer patients (TCGA) stratified for XRCC1 mRNA levels (cut at the median). **c** Kaplan-Meier analysis of the 5-year overall survival of lung cancer patients (TCGA) stratified for ALDH2 mRNA levels (cut at the median)

**Fig. 4:**
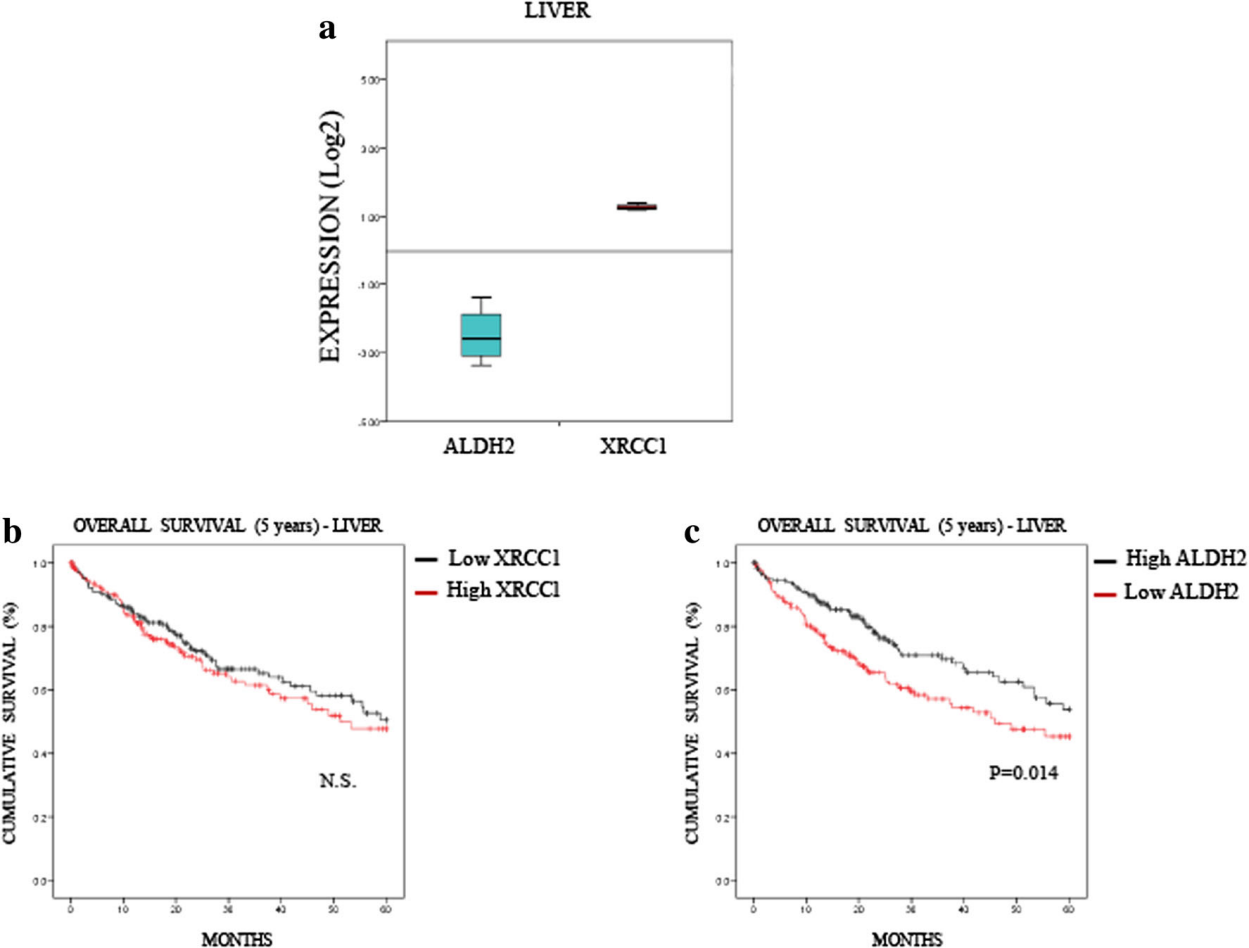
**Low ALDH2 expression predicts a poor prognosis in liver cancer. a** Boxplot representing the overall variation in XRCC1 and ALDH2 mRNA levels in liver cancers (Oncomine). **b** Kaplan-Meier analysis of the 5-year overall survival of liver cancer patients (TCGA) stratified for XRCC1 mRNA levels (cut at the median). **c** Kaplan-Meier analysis of the 5-year overall survival of liver cancer patients (TCGA) stratified for ALDH2 mRNA levels (cut at the median)

**Fig. 5:**
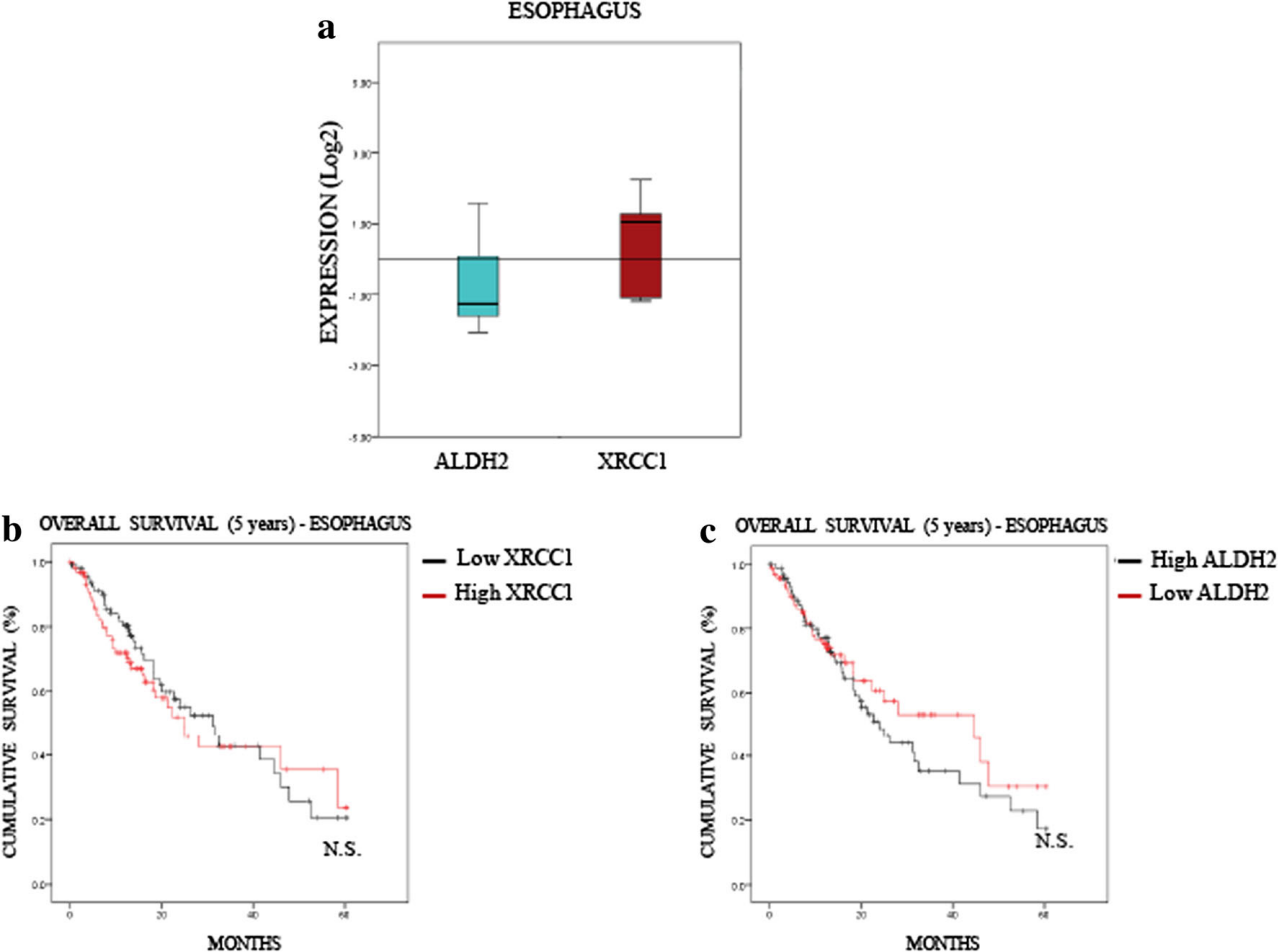
**ALDH2 and XRCC1 expression have no predictive value in oesophagus cancer. a** Boxplot representing the overall variation in XRCC1 and ALDH2 mRNA levels in oesophageal cancers (Oncomine). **b** Kaplan-Meier analysis of the 5-year overall survival of oesophageal cancer patients (TCGA) stratified for XRCC1 mRNA levels (cut at the median). **c** Kaplan-Meier analysis of the 5-year overall survival of oesophagus cancer patients (TCGA) stratified for ALDH2 mRNA levels (cut at the median)

To understand further the relevance of the interplay between XRCC1 and ALDH2 expression in cancer, we investigated whether the profile we observed in most cancers (i.e., low ALDH2 and high XRCC1 mRNA levels, Fig. 2) affects patient prognosis. To do so, we extracted provisional mRNA data from “The Cancer Genome Atlas” (TCGA) through the database cBioPortal [22, 23]. We selected data for lung (522 samples, Table S2) and liver (422 samples, Table S3) cancers in which the differences between ALDH2 and XRCC1 mRNA levels were pronounced (Figs. 3A and 4A) and compared these data to oesophageal cancer (186 samples, Table S4) in which XRCC1 and ALDH2 mRNA levels were very close (Fig. 5A). Looking at the overall survival rate at 5 years, we found that the XRCC1 mRNA level itself has no prognostic value in any of these cancers (Figs. 3B, 4B and 5B). On the contrary, we found that low ALDH2 mRNA levels, defined by separating values at the median, strongly predict a worse prognosis in lung and liver cancer (Figs. 3C and 4C). Interestingly, we found that the ALDH2 mRNA levels have no prognostic value in oesophageal cancer (Fig. 5C). Therefore, although XRCC1 mRNA levels alone have no prognostic value, low ALDH2 levels show a worse prognosis at 5 years, only in cancers showing a marked difference between ALDH2 and XRCC1 expression.

### XRCC1 levels or XRCC1/ALDH2 ratios further stratify the effect of low ALDH2 expression on 5-year overall survival

**Fig. 6:**
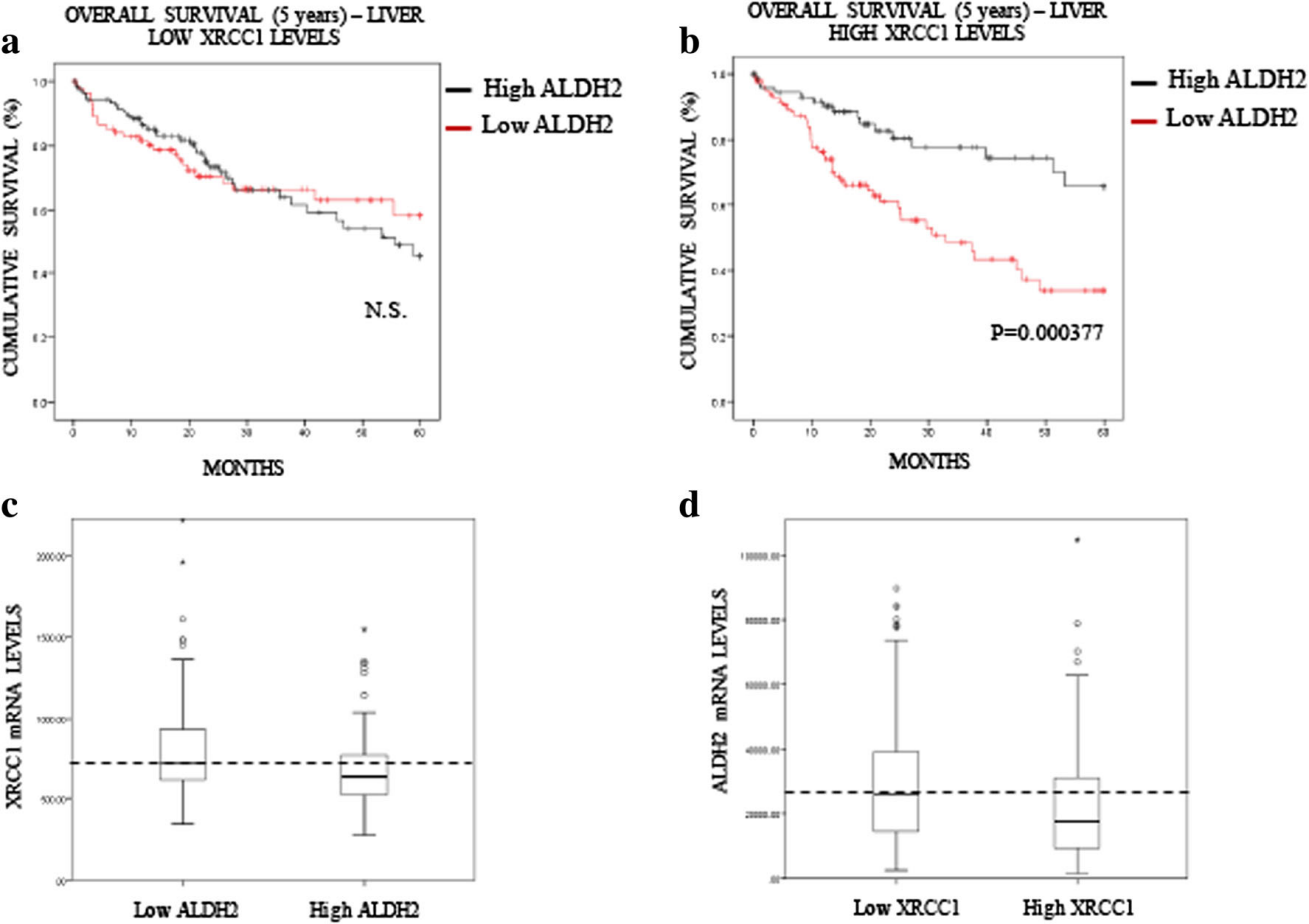
**XRCC1 levels increase the predictive value of ALDH2 expression in liver cancer. a** Kaplan-Meier analysis of the 5-year overall survival of liver cancer patients (TCGA) pre-stratified for low XRCC1 mRNA levels and stratified for ALDH2 expression (cut at the median). **b** Kaplan-Meier analysis of the 5-year overall survival of liver cancer patients (TCGA) pre-stratified for high XRCC1 mRNA levels and stratified for ALDH2 expression (cut at the median). **c** Boxplot representing the overall variation in XRCC1 mRNA levels in liver cancers (TCGA) related to ALDH2 expression (cut at the median). **d** Boxplot representing the overall variation in ALDH2 mRNA levels in liver cancers (TCGA) related to XRCC1 expression (cut at the median)

**Fig. 7:**
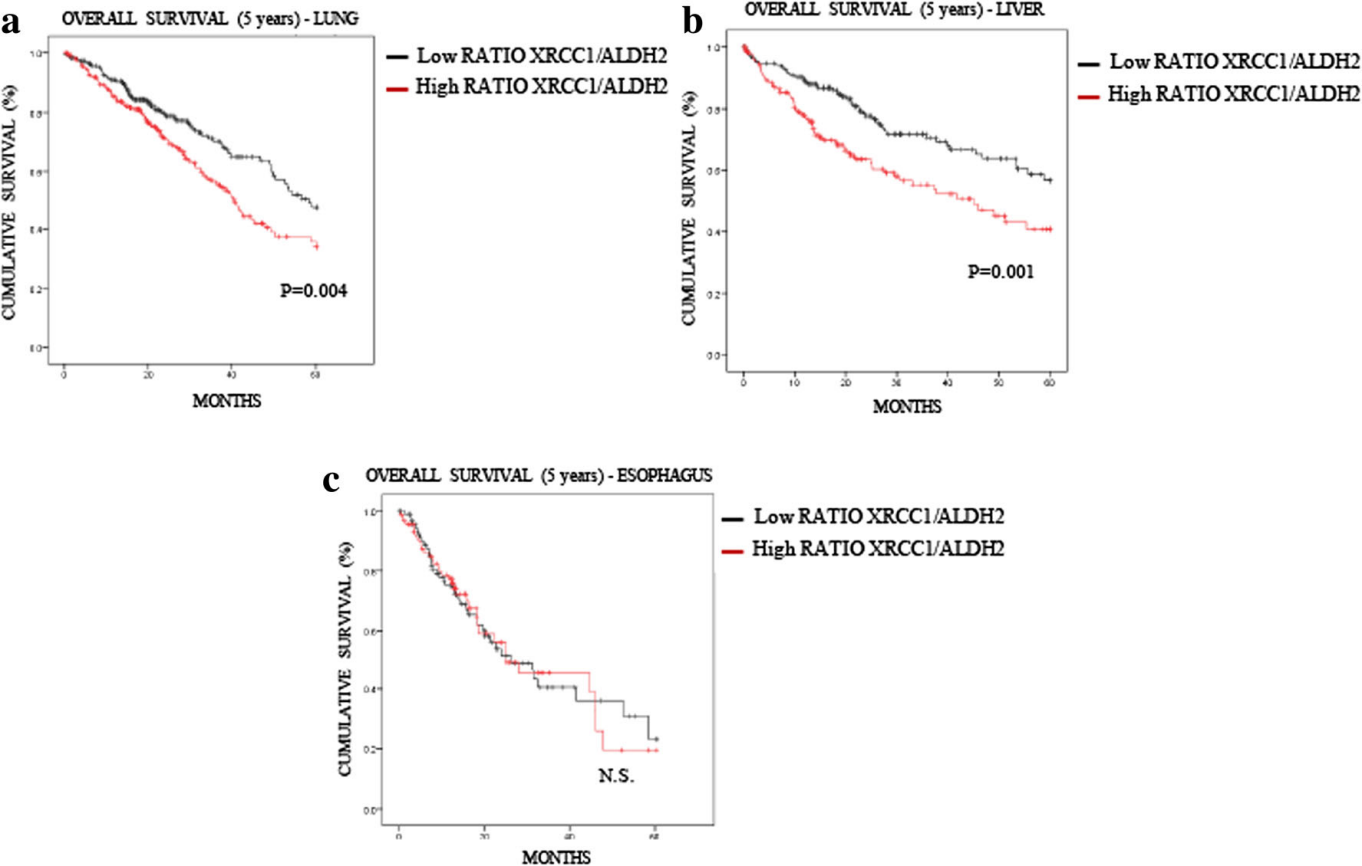
**XRCC1/ALDH2 mRNA level ratio has an increased predictive value compared to that of ALDH2 expression alone in lung and liver cancer patients. a** Kaplan-Meier analysis of the 5-year overall survival of lung cancer patients (TCGA) stratified for XRCC1/ALDH2 mRNA level ratio (cut at the median). **b** Kaplan-Meier analysis of the 5-year overall survival of liver cancer patients (TCGA) stratified for XRCC1/ALDH2 mRNA level ratio (cut at the median). **c** Kaplan-Meier analysis of the 5-year overall survival of oesophageal cancer patients (TCGA) stratified for XRCC1/ALDH2 mRNA level ratio (cut at the median)

In liver cancer patients, we observed a significant impact of ALDH2 mRNA expression on the overall survival rate, whereas the XRCC1 mRNA level itself had no predictive value (Fig. 4). Nevertheless, we stratified patients into two groups, low and high XRCC1 mRNA expression, and investigated whether this stratification improves the significance of ALDH2 mRNA expression on the prognosis. Strikingly, we found that the ALDH2 mRNA level has no prognostic value in the low XRCC1 mRNA expression group (Fig. 6A), whereas in the high XRCC1 mRNA expression group low ALDH2 mRNA expression was found to predict a very poor prognosis (Fig. 6B). Interestingly, we found that the XRCC1 mRNA levels are higher in the low ALDH2 mRNA samples (Fig. 6C) and that the ADLH2 mRNA levels are lower in the high XRCC1 mRNA group (Fig. 6D), confirming the above observed interplay between XRCC1 and ALDH2 expression (Fig. 1). In order to support the connection with the in vitro data of Fig. 1, we also used the APE1 and TDP1 mRNA levels to stratify ALDH2 expression and prognosis in liver cancer (Fig. S1). Interestingly, we found that APE1, whose depletion does not induce SSBs in their pure form [25], does not stratify the ALDH2 prognosis value (Fig. S1A and S1B). However, TDP1, whose depletion does mimic XRCC1 loss (Fig. 1F), was found to stratify the ALDH2 prognosis value in a similar fashion to XRCC1, although less powerfully (Figs. S1C and S1D). The POLβ levels did not show any prognostic value in liver carcinomas (data not shown). Next, we investigated whether the XRCC1 mRNA/ALDH2 mRNA expression ratio could also be used as a prediction factor. We found that the XRCC1 mRNA/ALDH2 mRNA ratio is significantly better at predicting the 5-year overall survival rate for both lung (Fig. 7A, compare to 3C) and liver (Fig. 7B, compare to 4C) cancers than ALDH2 levels alone. In oesophageal cancer, where the XRCC1 and ALDH2 mRNA levels show no difference (Fig. 5A), the XRCC1 mRNA/ALDH2 mRNA ratio shows no improvement in predicting the overall survival rate (Fig. 7C, compare to 5C). We thus conclude that XRCC1 mRNA levels along with ALDH2 mRNA levels may be used for stratification in order to predict the 5-year overall survival rate in liver and lung cancer patients and to refine their treatment strategy.

### Cancer-derived cell lines with low ALDH2 mRNA levels are sensitive to the Sp1 inhibitor Mithramycin A

**Fig. 8:**
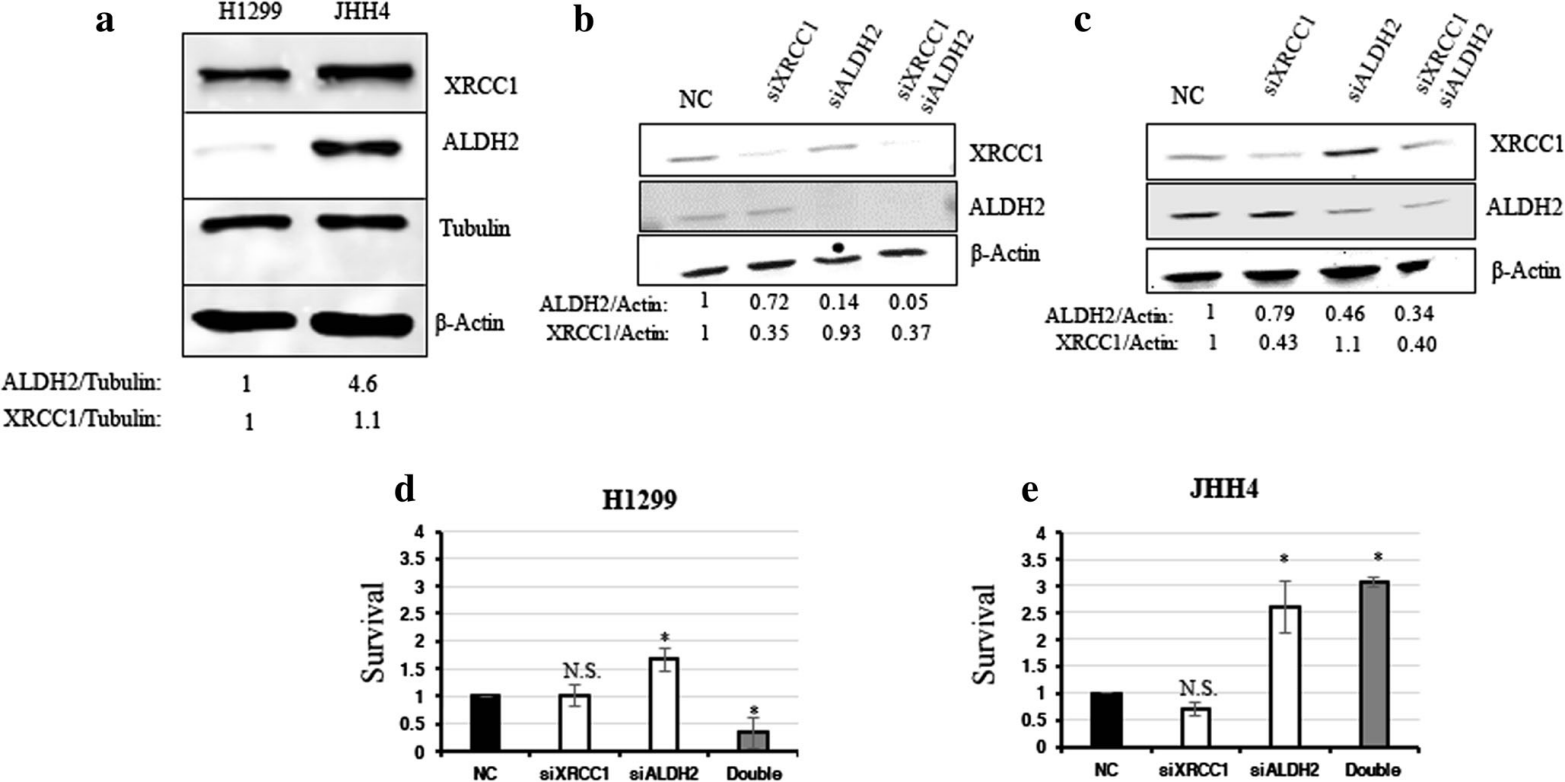
**Co-depletion of XRCC1 and ALDH2 sensitises low ALDH2-expressing cancer cells. a** Representative Western blots of ALDH2 and XRCC1 levels in H1299 and JHH4 cells. Densitometric quantifications of the data are presented below. **b** Representative Western blots of ALDH2 and XRCC1 levels in H1299 cells after 72 h of XRCC1 and/or ALDH2 knock down (KD). **c** Representative Western blots of ALDH2 and XRCC1 levels in JHH4 cells after 72 h of XRCC1 and/or ALDH2 KD. **d** Clonogenic survival analysis performed in H1299 cells after 72 h of XRCC1 and/or ALDH2 KD. **e** Clonogenic survival analysis performed in JHH4 cells after 72 h of XRCC1 and/or ALDH2 KD. Data are expressed as mean ± SD from at least three independent experiments * *p* < 0.05; N.S.: non-significant

**Fig. 9:**
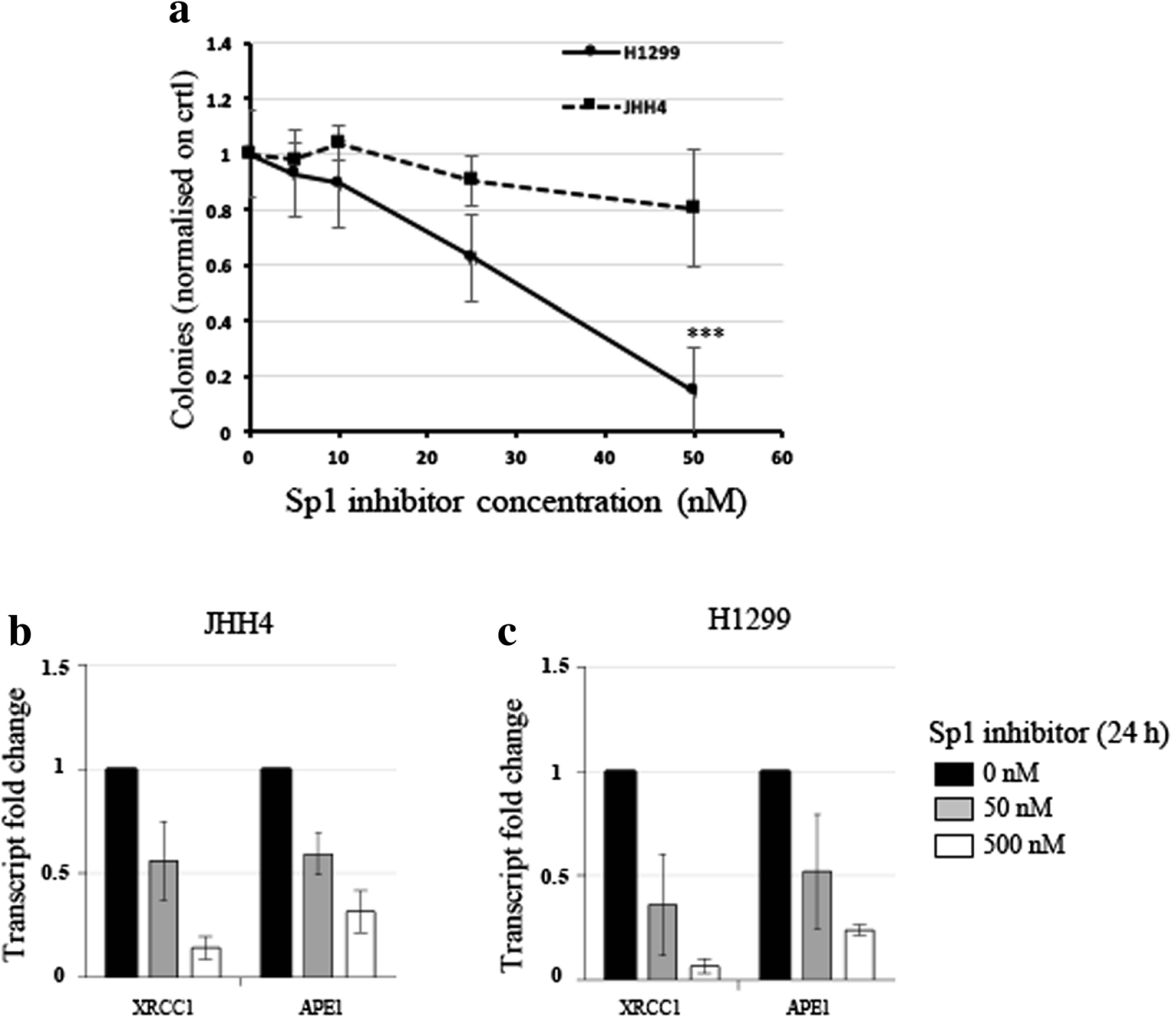
**Sp1 inhibitor Mithramycin A kills low ALDH2 expressing cancer cells. a** Clonogenic survival analysis performed in H1299 and JHH4 cells after two subsequent 24 h treatments (total 48 h) with Mithramycin A (Sp1 inhibitor). **b** qRT-PCR analysis of XRCC1 and APE1 mRNA levels in JHH4 cells after treatment with 50 nM or 500 nM Mithramycin A for 24 h. **c** qRT-PCR analysis of XRCC1 and APE1 mRNA levels in H1299 cells after treatment with 50 nM or 500 nM Mithramycin A for 24 h. qRT-PCR reference genes are B2M and GAPDH. Data are expressed as mean ± SD from at least three independent experiments. *** *p* < 0.001

We showed that XRCC1 and ALDH2 mRNA levels are related and that a high XRCC1 mRNA/low ALDH2 mRNA ratio seems to be accompanied by tumour aggressiveness. We then wondered whether destabilising this balance could have a deleterious effect on cancer cell survival. To do so, we selected two cancer-derived cell lines, i.e., H1299, a lung cancer-derived cell line with a low ALDH2 expression and JHH4, a liver cancer-derived cell line with a high ALDH2 expression (Fig. 8A). We then proceeded to knock down XRCC1 mRNA expression, ALDH2 expression, or both, and investigated cellular survival using a clonogenic assay. We found that H1299 cells (ALDH2 low), do not tolerate decreases in both XRCC1 and ALDH2 proteins (Fig. 8B and D, last column), whereas JHH4 cells (ALDH2 high) cope better after a double knockdown of XRCC1 and ALDH2 (Fig. 8C and E, last column), most probably because they do not completely lose ALDH2 expression after knockdown (Fig. 8C). Interestingly, we found that ALDH2 knockdown alone promotes higher survival rates for both the H1299 (Fig. 8D, column 3) and JHH4 (Fig. 8E, column 3) cells, supporting our observation that lower ALDH2 levels are advantageous for cancer cells (Fig. 2). We also found that single knockdown of XRCC1 does not reduce the survival of H1299 cells (Fig. 8D, second column) even if their level of ALDH2 is already low. We believe that this may be due to there being some BER activity remaining as a result of incomplete siRNA knockdown of XRCC1 expression [25]. We thus hypothesise that H1299 cells may be sensitised by a global shutting down of the BER pathway. We have recently uncovered that inhibition of the Sp1 transcription factor decreases the expression of the BER protein APE1 [25], as well as other members of this pathway [30]. We, therefore, set out to investigate the survival of H1299 and JHH4 cells after treatment with the Sp1 inhibitor Mithramycin A. We found that H1299 cells are extremely sensitive to very low doses of Mithramycin A, whereas JHH4 cells are resistant (Fig. 9A). We also confirmed a decreased expression of XRCC1 and APE1 in JHH4 (Fig. 9B) and H1299 (Fig. 9C) cells after Sp1 inhibition by qRT-PCR. Because Sp1 regulates the whole BER pathway, Sp1 inhibition seems more potent in killing H1299 cells than depletion of XRCC1 alone. These data represent a proof of principle that, although low expression of ALDH2 provides cancer cells with some growth advantage (Fig. 8D and E), they may still be dependent on an efficient BER pathway (Fig. 9A). In conclusion, we found that several types of cancer show a profile with a lower ALDH2 and a higher XRCC1 expression that is linked to a poor prognosis. Cancer cells with this profile could potentially be targeted by using BER enzyme inhibitors or by shutting down the pathway through BER gene expression inhibition.

## Discussion

Metabolic changes are a cornerstone of cancer development [31, 32]. Recent scientific advances in the understanding of cancer metabolism have shown the importance of defining the pathways that may limit cancer progression with the hope of designing reliable clinical strategies with benefits for the patients [32]. In this context, the pathways to be targeted must represent unique avenues taken by cancer cells to boost their proliferation and survival. By using these particular metabolic pathways, cancer cells leave traces or clues in the form of specific waste products that can be used to trace new metabolic pathways used by them [33]. BER is one of the first DNA repair pathways deregulated during cancer progression [25]. Here, we used XRCC1 levels as a readout for BER efficiency. This is because XRCC1 depletion is a model known for the purity of the type of lesion it causes, compared to APE1 depletion, and for the robust BER deficiency it induces, compared to POLβ depletion [17, 25, 26]. We have previously shown that loss of XRCC1 triggers metabolic changes resembling those found in many cancers [17, 34]. Here, we hypothesised that alterations in aldehyde metabolism could be part of these changes. Recently, aldehydes such as AcAl, methylglyoxal and formaldehyde have generated significant interest for their role as waste products of cancer metabolism [2, 33, 35, 36]. Interestingly, aldehyde production, when linked to beneficial metabolic pathways for cancer, acts as a double-edged sword associated with high genotoxicity [33, 35]. Cancer cells rely on detoxification enzymes such as ALDH2 that can neutralise aldehydes and keep a semblance of metabolic equilibrium [5, 29, 37]. Nevertheless, cancer cells frequently exhibit an excess of aldehydes and, therefore, a high occurrence of aldehyde-related DPCs and genetic instability [2, 9, 28]. Surprisingly we observed in this study, by using bioinformatics analyses, that most cancers, especially lung and liver cancers, gamble with lowering their expression of ALDH2 along with overexpressing XRCC1. This observation correlates with the growing body of evidence indicating a high occurrence of DPCs and their link to genetic instability in cancer [27, 28]. Interestingly, DPC accumulation in the liver is particularly oncogenic as it has been found that patients lacking the fundamental DNA-protein crosslink repair metalloprotease SPRTN/DVC1 develop very early onset aggressive hepatocellular carcinomas [38]. Indeed, we show that inhibition of ALDH2 increases the occurrence of DPCs and that low expression of ALDH2 is associated with a poor prognosis for liver cancer patients. Surprisingly, despite the existence of different ALDH isoforms, we found that only ALDH2 expression has an impact on liver cancer (data not shown). This would suggest a fundamental function of ALDH2 in aldehyde detoxification, superior to any other ALDH.

Several non-exclusive hypotheses can be put forward for a putative role of BER in the control of aldehyde-related damage. Firstly, our data support the idea that BER is involved directly in the repair of aldehyde-related damage. Aldehydes attack DNA bases and these alterations can be repaired by BER [39]. In addition, it has been proposed that BER may be involved in the repair of DNA inter-strand crosslinks that can also be caused by aldehydes [40, 41]. Finally, although there are very little data linking BER to the repair of DPCs, it is interesting to note that XRCC1 is involved in the removal and repair of lesions caused by trapped TOP1, and so is TDP1 [42]. If the TOP1 removal mechanism shares any similarity to DPC repair, this could potentially place BER downstream of SPRTN/DVC1 after the removal of the crosslinked protein. Altogether, this could mean that the BER pathway may be involved in the repair of aldehyde-related DPCs and this, in turn, could explain why low ALDH2 expressing cancer cells have increased BER levels. Secondly, our data could be interpreted by using an alternative scenario, seeing loss of BER as a cause of aldehyde-related toxicity. We have previously shown that the loss of BER triggers metabolic changes, notably by increasing the activity of the one-carbon cycle [17]. More recently, it has been shown that endogenously produced formaldehyde plays a major role in the one-carbon cycle [2]. If BER-deficient cells increase the activity of this cycle, it is reasonable to suggest that this would, in turn, increase the concentration of formaldehyde in the cell as both a waste product and a reaction intermediate. Interestingly, we previously found that BER-deficient cells exhibit increased levels of the anti-oxidant GSH [20]. It is known that GSH can be used as an alternative to detoxify aldehydes [2]. Therefore, we propose that BER-deficient cells increase ALDH2 expression in order to prevent aldehyde-induced DNA damage. This would mean that low ALDH2 expressing cancer cells are at a high risk of aldehyde overload if BER levels are not high enough. Thirdly, we could envisage that BER-deficiency, by creating numerous SSBs, some of which may contain aldehydes at the end of the strand break [42], can easily cause DPCs [26]. This hypothesis is reinforced by the fact that the two models used in this study, XRCC1 and TDP1 depletion, have in common a very substantial accumulation of SSBs. Finally, whatever the role of BER, knowing that any or all these hypotheses could be correct, we propose as proof of principle that cells may not survive by losing both BER and ALDH2 activities. By inhibiting Sp1, one of the main regulators of BER protein expression [25], we observed a higher sensitivity for BER loss in cells expressing low levels of ALDH2. Although Mithramycin A is quite toxic in vivo [43] and cannot be used to treat patients directly, the development of less toxic Mithramycin A derivatives or the use of other BER inhibitors [44–46] may open up a new avenues to target specific metabolic defects employed by certain cancer cells.

In conclusion, we have discovered a previously unknown interconnection between the BER pathway and aldehyde detoxification. We demonstrated the importance of this relationship in the prognosis of liver and lung cancer and suggest that these observations may pave a new way towards targeting cancer metabolism.

## Electronic supplementary material

Table S1 (XLSX 16 kb)

Table S2 (XLSX 52 kb)

Table S3 (XLSX 74 kb)

Table S4 (XLSX 24 kb)

Figure S1 (PNG 869 kb)

High resolution image (TIF 119 kb)
