# Supplementary figures and images for: Interplay between base excision repair protein XRCC1 and ALDH2 predicts overall survival in lung and liver cancer patients
Source: Cell Oncol (Dordr). 2018 Aug 7;41(5):527–39. doi: 10.1007/s13402-018-0390-8 (PMC6153960; doi:10.1007/s13402-018-0390-8)

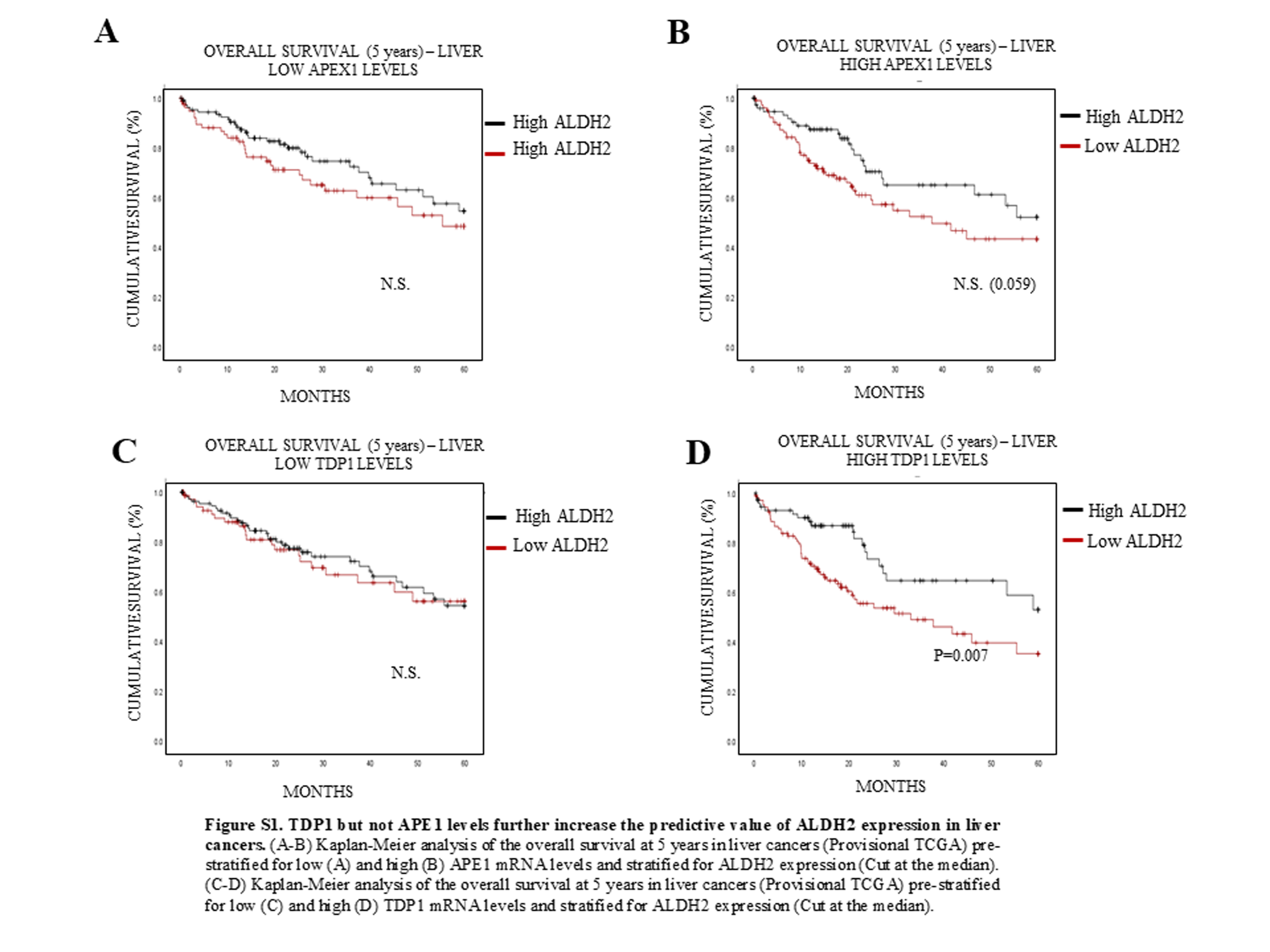

Supplement: Supplementary file 5 — (PNG 869 kb) [file 13402_2018_390_Fig10_ESM.png]

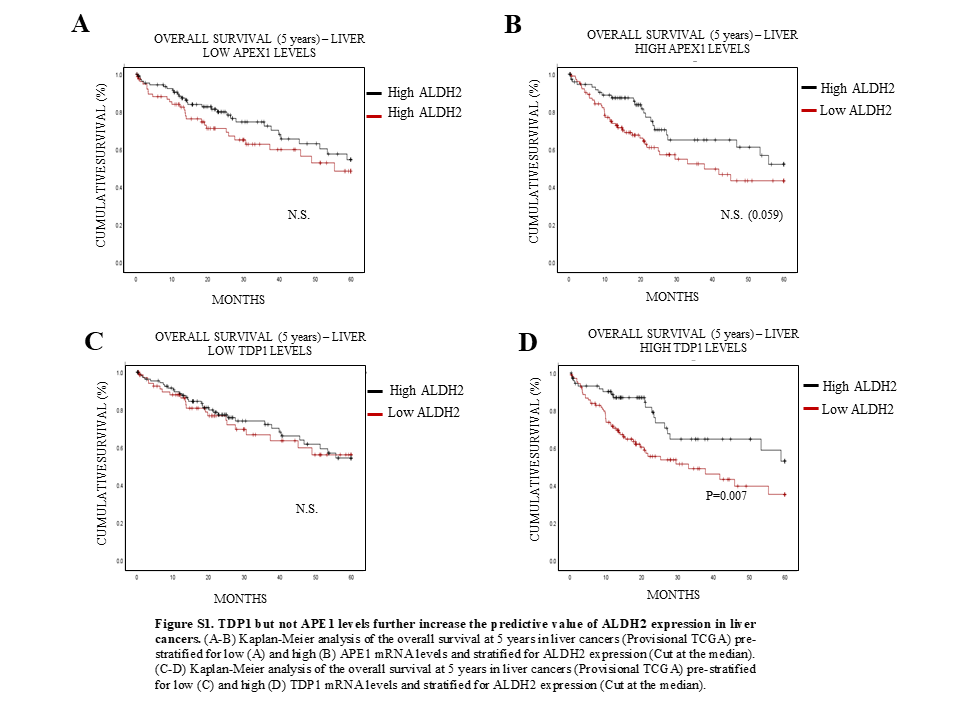

Supplement: Supplementary file 6 — High resolution image (TIF 119 kb) [file 13402_2018_390_MOESM5_ESM.tif]
